# Editorial: Primary cilia as therapeutic targets

**DOI:** 10.3389/fmolb.2023.1322873

**Published:** 2023-10-26

**Authors:** Yuhei Nishimura, Masaki Saito, Wataru Otsu, Keiko Miyadera

**Affiliations:** ^1^ Department of Integrative Pharmacology, Mie University Graduate School of Medicine, Tsu, Mie, Japan; ^2^ Mie University Research Center for Cilia and Diseases, Tsu, Mie, Japan; ^3^ Department of Molecular Physiology and Pathology, School of Pharma-Sciences, Teikyo University, Tokyo, Japan; ^4^ Department of Biomedical Research Laboratory, Gifu Pharmaceutical University, Gifu, Japan; ^5^ Department of Clinical Sciences and Advanced Medicine, School of Veterinary Medicine, University of Pennsylvania, Philadelphia, PA, United States

**Keywords:** primary cilia, atopic dermatitis, inherited retinal degeneration, cancer, obesity, preeclampsia, gene therapy, therapeutic target

The primary cilium is an antenna-like structure of the plasma membrane. Primary cilia contain receptors and channels that detect extracellular signals and transduce them into the cells to regulate physiological functions such as cell proliferation, differentiation, and migration. The formation of primary cilia is dynamically regulated depending on the context. Dysregulation in the signaling and formation of primary cilia can lead to various diseases. The physiological and pathophysiological mechanisms underlying the regulation of primary cilia have been extensively investigated. These studies have revealed many aspects of primary cilia which can be considered as therapeutic targets. The scope of this Research Topic is to integrate the current knowledge and opinions of primary cilia in relation to their potential as therapeutic targets.

Toriyama et al. analyzed the primary cilia of human dendritic cells (DCs) and Langerhans cells (LCs). They revealed that primary cilia mediated the signaling of granulocyte-macrophage colony-stimulating factor, a Th2 cytokine, to stimulate the proliferation of DCs and that the formation of primary cilia is halted in mature DCs. They also found that the primary cilia of LCs and keratinocytes from patients with atopic dermatitis were aberrantly ciliated in immature and proliferating states. They proposed a vicious cycle in atopic dermatitis in which ciliated immature DCs strongly recognized antigens and secreted GM-CSF to stimulate the proliferation in autocrine and paracrine fashions. This situation might be the same as some groups of medulloblastoma where primary cilia aberrantly mediate oncogenic signaling (Youn et al., 2022). Suppression of the aberrant signals through primary cilia may be a novel therapeutic approach for these disorders.

Takahashi et al. characterized MAP9, a microtubule-binding protein, as a modifier in cone-rod dystrophy caused by a primary mutation of the retinitis pigmentosa GTPase regulator interacting protein 1 (*RPGRIP1*) in a canine model. MAP9 is thought to stabilize the ciliary microtubule-axoneme of photoreceptors and to regulate motor-protein-mediated ciliary trafficking (Monroy et al., 2020; Tran et al., 2023). Takahashi et al. studied the homozygous 22 kb deletion in canine *MAP9* which causes only a slight amino acid difference from wild-type MAP9 due to a duplication. They demonstrated that mutant MAP9 exacerbated the photoreceptor degeneration in RPGRIP1 mutants both functionally and structurally. They identified that MAP9 is prominently localized in the basal body of primary cilia suggesting an important role in maintaining the structure of ciliary microtubule axoneme. Their findings highlighted the role of MAP9 as a modifier and that the RPGRIP1 complex may be a therapeutic target in the cilia-related retinal degeneration.

Obesity is caused by various factors, including adipogenesis in response to high-fat diet and the dysregulation of metabolic homeostasis. Primary cilia are involved in both the peripheral adipogenesis and the central control of metabolism associated with obesity (Engle et al., 2021; Hilgendorf, 2021). For example, the formation of primary cilia changes dynamically during the differentiation of preadipocytes into adipocytes while the differentiation is suppressed in preadipocytes with knockout of trichoplein, a suppressor protein for ciliogenesis, in which the primary cilia are overly elongated (Yamakawa et al., 2021). The expression of alpha-melanocyte stimulating hormone (α-MSH), a neuropeptide to suppress appetite, is stimulated by leptin through the leptin receptor located around the primary cilia of proopiomelanocortin (POMC)-expressing neurons in the arcuate nucleus. Mutations of BBS1 or BBS10 that cause Bardet-Biedle syndrome, a ciliopathy characterized by hyperphagic obesity, abnormally elongate the primary cilia of the POMC-expressing neurons and inhibit the α-MSH-mediated suppression of appetite (Wang et al., 2021). Ciliary-specific signaling in other hypothalamic regions, such as the paraventricular nucleus and lateral hypothalamus, is also involved in leptin-mediated energy homeostasis. DeMars et al. provide a comprehensive review of neuronal primary cilia regulating metabolic homeostasis in these inter-neuronal connectivity. These studies may pave the way for novel anti-obesity therapies by correcting the abnormal signaling related to primary cilia.

The relationship between primary cilia and the signaling modulated by cholesterol and lipid rafts has attracted increasing attention in areas of research, especially those focusing on cancer (Radhakrishnan et al., 2020; Nishimura et al., 2021). Kimura et al.elegantly review the association between cholesterol and ciliary signaling such as Sonic hedgehog and Wingless/INT pathways, suggesting that enzymes involved in cholesterol synthesis such as hydroxymethylglutaryl-CoA reductase and hydroxysteroid 11-β dehydrogenase 2 can be therapeutic targets against cancer. Hart provides an interesting hypothesis that increased dietary polyunsaturated fat may suppress ciliogenesis which could be the cause of preeclampsia, a complication of pregnancy characterized by hypertension, protein urea, and other signs of organ damage. Cholesterol and lipid rafts have been considered important therapeutic targets in various diseases (Sviridov et al., 2020; Vona et al., 2021). Examining the involvement of primary cilia provides novel insights into the therapeutic approaches targeting cholesterol and lipid rafts.

Saito et al. summarize recent advances in the understanding of cilia mechanisms and their applications as therapeutic targets, especially focusing on ciliary receptors, ciliogenesis, intracellular trafficking pathway to primary cilia, and animal models and clinical trials in inherited retinal diseases. These studies suggest that dysregulation of primary cilia associated with diseases may be treated by genetic and/or pharmacological therapies targeting genes and their protein products as well as signal transductions related to the primary cilia. We may change the functions of primary cilia in a specific cell type to modulate other cellular components dysregulated in diseases.
